# Imaging the developing brain with near-infrared spectroscopy in cochlear implanted children

**DOI:** 10.1162/IMAG.a.90

**Published:** 2025-08-01

**Authors:** Gaia Lucarini, Caroline Nallet, Davide Brotto, Valeria Del Vecchio, Alessandro Martini, Patrizia Trevisi, Anna Rita Fetoni, Judit Gervain

**Affiliations:** Department of Developmental and Social Psychology, University of Padua, Padua, Italy; Padova Neuroscience Center, University of Padua, Padua, Italy; Section of Otolaryngology, Department of Neuroscience DNS, University of Padua, Padua, Italy; Hearing and Balance Unit, Department of Head and Neck, Federico II University Hospital, Naples, Italy; Audiology Section, Department of Neuroscience and Reproductive Sciences and Dentistry, University of Naples Federico II, Naples, Italy; Integrative Neuroscience and Cognition Center, Université Paris Cité & CNRS, Paris, France

**Keywords:** cochlear implant, functional near-infrared spectroscopy (fNIRS), children, brain development, critical period, neuroplasticity

## Abstract

Cochlear implants (CIs) have revolutionized how we treat hearing impairment. Despite major technological and clinical advances, some CI children’s language abilities remain below those of their age-matched peers, and there is still considerable individual variability in final outcomes. One important factor underlying this may be individual differences in brain plasticity before and after implantation. However, the neural changes induced in the developing brain by deafness, language deprivation, and the restoration of hearing due to implantation are little understood, in part because the methodological options available are limited. Recently, functional near-infrared spectroscopy (fNIRS) has emerged as a fully CI-compatible, infant-friendly, non-invasive, and inexpensive technique that holds the promise of shedding light on the neural mechanisms accompanying deafness and CI use. Here, we review the existing fNIRS studies with developmental populations. We then discuss the methodological challenges that using fNIRS with CI children raise. Finally, we describe open questions that fNIRS has the potential to answer. We conclude that fNIRS is a powerful tool to investigate the neural mechanisms and changes brought about by deafness and the subsequent restoration of hearing with CI.

## 1 Introduction

Cochlear implants (CIs) have revolutionized how we treat hearing impairment. A CI is a neuroprosthetic device that is surgically implanted into the cochlea to provide direct electrical stimulation to the auditory nerve. Since the 1970s, cochlear implants have been applied in an ever-increasing number of patients, with age of implantation radically decreasing. Currently, children are often implanted as early as 6–12 months of age with the aim of reducing auditory and language deprivation, as speech perception and language development are known to have critical periods early in life (Franchella et al., 2024; Werker & Hensch, 2015), thus deprivation has a detrimental impact on later language and academic outcomes (Castellanos et al., 2018; Dikderi et al., 2023). Indeed, early implantation, especially before age 3–4 years, has been shown to lead to better overall outcomes (Kral & Sharma, 2012; Sharma & Dorman, 2011).

Yet, despite these advances, the language abilities of many CI using children remain below those of their age-matched peers. In general, individual variability in final outcomes is considerable, even among children implanted early in life (Arjmandi et al., 2022). The reasons for this variability are only partially understood. Some factors such as duration of deafness, age at implantation, age at onset of deafness, length of implant use, cognitive skills, communication mode, the surgical procedures used, and the characteristics of the device (e.g., number and placement of electrodes) have already been explored (Arjmandi et al., 2022; Peterson et al., 2010). Here, we explore the possibility that residual auditory perception and brain plasticity/reorganization before and after cochlear implantation may be another important factor in explaining individual variability in CI outcomes. These factors, however, have so far remained relatively unexplored, in part due to the methodological challenges of testing young infants’ perceptual and brain development. Infant brain imaging is challenging even in typically developing infants. CIs add further complexity, as they create electric noise and interference with EEG and MRI recordings.

In the last decades, functional near-infrared spectroscopy (fNIRS) has emerged as a new imaging technique particularly well suited for developmental populations (Ayaz et al., 2022; Gervain et al., 2023). fNIRS is infant-friendly and fully compatible with CIs (Bortfeld, 2019; Ferrari & Quaresima, 2012), offering an unprecedented opportunity to assess the neural changes that result from deafness, language deprivation, and the restoration of hearing with CI. fNIRS uses red and infrared light to measure the concentration changes of oxygenated and deoxygenated hemoglobin in the brain associated with focal brain activity. Unlike EEG, fNIRS does not directly measure neural activity, but rather its hemodynamic correlates, similarly to the BOLD response measured by fMRI. Consequently, its temporal resolution is not high, but not being subject to the inverse problem present in EEG, its spatial localization is accurate, albeit lower than that of MRI. Since the depth of penetration of the light into the head and thus the depth of the cortical volume that fNIRS can probe depends on the thickness of the tissues around the brain (e.g., skin, skull etc.), fNIRS is well suited for brain imaging in infants and children, whose thinner tissues allow deeper penetration into the cortex. As NIR light does not interact with the electric signals produced by the CI, the two devices are fully compatible. These two features thus make fNIRS the method of choice for investigating neural reorganization in developmental CI populations.

Yet, while several studies used fNIRS to investigate adults with CI (Anderson et al., 2019; Anderson, Lazard, et al., 2017; Anderson, Wiggins, et al., 2017; Esmaelpoor et al., 2025; Fullerton et al., 2023; Olds et al., 2016; X. Zhou et al., 2018), studies with implanted infants are mostly lacking (Harrison & Hartley, 2019). Only a few studies investigated the neural reorganization resulting from hearing loss and subsequent cochlear implantation in very young infants with fNIRS (Coëz et al., 2022; Wang et al., 2021), although somewhat more studies exist with older children (e.g., Sevy et al., 2010). This knowledge gap is critical both from a theoretical as well as a clinical perspective. Theoretically, the neural changes induced by auditory and language deprivation and the subsequent restoration of hearing due to CI provide important insights into mechanisms of brain plasticity and critical periods. From a clinical point of view, a better understanding of these mechanisms has the potential to offer new therapeutic and intervention strategies, leading to better outcomes for developmental CI patients.

In this paper, we will therefore first review the fNIRS studies targeting deaf and hard-of-hearing (DHH) children before and after cochlear implantation to get an overview of the existing empirical evidence. To identify the relevant publications, we searched PubMed and Google Scholar using the following key words as search terms: NIRS, deafness, children, and synonyms. We restricted our search to articles published between 2001 and 2025. This search has yielded 6100 studies. After checking for duplicates, papers written in English and assessing relevance on the basis of the title and abstract, 17 studies were retained for the review. Table 1 describes the main characteristics of each study. We then grouped together studies that used similar experimental paradigms, and addressed similar research questions. These are discussed in separate sections below. First, we present studies that used only auditory stimuli to understand how DHH children’s brains processes auditory stimuli (Y. Chen et al., 2022; Z. Chen et al., 2024; Coëz et al., 2022; Sevy et al., 2010; Wang et al., 2021). Then, we address neural reorganization due to cross-modal plasticity by reviewing studies using combined auditory and visual stimuli (Alemi et al., 2023; Mushtaq et al., 2020; X.-Q. Zhou et al., 2023) or visual-only stimuli (Alemi et al., 2024; Deroche et al., 2024). Lastly, we describe studies investigating functional connectivity and networks at rest or during auditory stimulation (Calmels et al., 2022; G. Liu et al., 2023; Y. Liu et al., 2023; Tan et al., 2024; Wang et al., 2022; Wu et al., 2024, 2025).

**Table 1.** Summary table of included articles.

Author(s)	N* (age)	Hearing loss	Experimental Paradigm			fNIRS characteristics
Alemi et al. (2023)	50 HL, CI 25 NH (7–18 years)	Profound HL Bilateral CIs (except 4 subjects with unilateral CI) AoI: < 4 years Diagnosed age not specified (first HA: 1–33 months)	AV	Audio-visual integration task:ba/ and /ga/ syllables presented visual-only (V), auditory-only (A), and congruent auditory-visual (AV) modes	EEG: event related design fNIRS: block design	122 channels NIRx NIRScout system
Alemi et al. (2024)	50 HL, CI 25 NH (7–18 years)	Profound HL Bilateral CIs (except 4 subjects with unilateral CI) AoI: < 4 years Diagnosed age not specified (first HA: 1–33 months)	O	Motor task eliciting both motor and somatosensory processing	Block design	122 channels NIRx NIRScout system
Calmels et al. (2022)	Expected: 30 HL and 30 NH (5-16 years)	Expected SSD	A	Study protocol: environmental sounds, human voice (non-speech), speech sounds, white noise	Block design	18 channels – auditory cortex and the superior temporal area System not specified
Y. Chen et al. (2022)	21HL, CI25 NH (5-10 years)	Pre-lingual deafness Right CI only AoI: 3; 1 (1; 5–9; 7)	A	Auditory task: strong-prosodic (SP) vs. weak-prosodic (WP) sentences	Event related design	20 channels – bilateral temporal cortex, inferior frontal cortex, and inferior parietal cortexLIGHTNIRS
Z. Chen et al. (2024)	47 HL, CI(35.47 ± 17.24 months)	Severe-to-profound HL Onset of HL not specified 46 unilateral CI and 1 bilateral CIs AoI: 12–48 months	A	Auditory task: passive listening to speech, noise, speech in noise, and music	Block design	20 channels—bilateral auditory and language-related areas System not specified
Coëz et al. (2022)	8 HL 9 NH Pre-CI: 12 months Post-CI: 24 months (no data)	Profound HL Diagnosed age not specified Pre-CI: HA Post-CI: CI (no data)	A	Auditory task: voice stimuli (words, non-words, foreign language, laughs, sighs, various onomatopoeia), non-voice stimuli (natural sounds, animals, modern human environment, and musical instruments)	Block design	30 channels—auditory areas in the bilateral temporal, frontal and parietal areas NIRx NIRScout system
Deroche et al. (2024)	50 HL, CI 25 NH (7–18 years)	Profound HL Bilateral CIs (except 4 subjects with unilateral CI) AoI: < 4 years Diagnosed age not specified (first HA: 1–33 months)	V	Visual task: rotating circular checkerboard	EEG: event-related design fNIRS: block design	122 channels NIRx NIR Scout system
G. Liu et al. (2023)	55 HL 60 NH (3-9 months)	Congenital mild-to-profound HL	RS	Resting state	Resting state	64 channels—frontal, temporal, parietal, and occipital areas NirSmart system
Y. Liu et al. (2023)	36 HL 12 NH (3–10 months)	Congenital SSD (18) or mild-to-moderate UHL (18)	RS	Resting state	Resting state	64 channels—frontal, temporal, parietal, and occipital areas NirSmart system
Mushtaq et al. (2020)	19 HL, CI 20 NH (6–12 years)	Congenital or early-onset HL, bilateral CIs activated at 27.4 months (range 10–86 months)	AV	Visual task: visual speech stimulus only (lip-reading) Auditory task: normal speech, Signal-correlated noise (SCN), steady speech-shaped noise (SSSN)	Event-related design	24 channels—temporal areas Hitachi system
Sevy et al. (2010)	37 HL, CI > 4 mo 9 HL, CI activation 11 NH children (2-19 years)11 NH adults	Profound HL Diagnosed age, AoI, CI side not specified	A	Auditory task: sentences from a story	Block design	4 channels—temporal lobe and superior temporal gyrus NIRS 2CE machine (TechEn)
Tan et al. (2024)	22 HL (4 months) 28 NH (1–2 days) 15 NH (4 months)	Congenital HL > 40 dB	RS	Resting state	Resting state	36 channels—frontal, temporal, and parietal cortices NirSmart system
Wang et al. (2021)	22 HL (13–30 months) Pre-CI: HA Post-CI: CI + controlateral HA	Prelingual severe-to-profound HL Unilateral CI AoI: 22.5 months (13–38)	A	Auditory task: passive listening to pseudo-sentences with different emotional contents (fear, angry, happy, neutral)	Block design	52 channels—temporal, frontal, and central regionsNirSmart system
Wang et al. (2022)	34 HL, CI (36.82 ±17.65 months) Tested before and after CI 35 NH adults	Prelingual severe-to-profound HL 15 left CI 19 right CI AoI: 32.73 (15.43)	RS, A	Resting state Auditory task: passive listening to speech, music, and noise	Resting state and block design	20 channels—temporal lobe and adjacent language processing areas NIRx NIRSport2
Wu et al. (2025)	84 HL, CI (3.02 ± 2.17 years) 35 NH adults	Prelingual severe-to-profound HL AoI: 38.75 (25.59) 6 bilateral CI, the rest unilateral	RS, A	Resting state Auditory task: passive listening to speech, music, noise, and speech in noise	Resting state and block design	20 channels—bilateral temporo-parieto-frontal auditory and language-related areasNIRx NIRSport2
Wu et al. (2024)	67 HL, CI (3.02 ± 2.17 years) 35 NH adults	Prelingual severe-to-profound HL AoI: 33 (16) 5 bilateral CI, the rest unilateral	RS, A	Resting state Auditory task: passive listening to speech, music, noise, and speech in noiseHere analyzed only speech and noise conditions	Resting state and block design	20 channels bilateral temporo-parieto-frontal auditory and language-related areasNIRx NIRSport2
X.-Q. Zhou et al. (2023)	43 HL 41 NH (6–9 years)	Prelingual HL (0–28 months as onset time) Right CI only AoI: 2.9 (1–5.12)	AV	Visual task: visual speech only Auditory task: speech in quiet (SIQ), speech in noise (SIN)	Block design	44 channels—temporal regions Hitachi system

* Reported only N subjects who completed testing/provided enough data for the analysis.

Abbreviations:

Hearing Loss: AoI – Age of Implantation; CI – Cochlear Implant; HA – Hearing Aid; HL – Hearing Loss; NH – Normal Hearing; pre-CI – data collected before CI surgery; post-CI - data collected after CI surgery and activation; SSD - Single-Side Deafness; UHL – Unilateral Hearing Loss.

Experimental paradigm: A – Auditory task; V – Visual task; O – Others; RS – Resting State.

We note from the outset that studies differ widely in terms of sample size, age of participants, type and severity of hearing loss, treatment, CIs characteristics, age of implantation, as well as the fNIRS system used and the brain area targeted (see Table 1). Accordingly, one of the main objectives of the current review is to highlight the lack of systematic research on the plastic changes in neural organization related to auditory and language deprivation and restoration due to deafness and cochlear implantation. The paper then argues that one possible solution to this problem is the use of fNIRS with the aim of furthering our theoretical knowledge and providing possible clinical applications.

## 2 What Does fNIRS Tell Us about the Developing Brain before and after Cochlear Implantation?

### 2.1 The neural correlates of auditory and speech processing in deaf and hard-of-hearing children before and after CI as measured by fNIRS

In 2010, Sevy and colleagues conducted a landmark study, aiming at determining if fNIRS was a reliable tool to measure the activity of the auditory cortex in response to speech stimuli in 2 19-year-old children with cochlear implants (Sevy et al., 2010). To do so, they first recorded the activity of the auditory cortex in a group of three normal-hearing adults in response to speech, specifically a female voice reading a story, using fMRI. Then, they compared the fMRI data with fNIRS data by testing the same adults and eight additional participants, using the exact same stimuli. They found that the two techniques revealed similar responses, both highlighting a significant hemodynamic response localized in the superior temporal gyrus. The same procedure was then applied to eight additional NH adults and to three different populations of children: normal-hearing children, deaf children with at least 4 months of experience with hearing through the CI, and deaf children who were tested on the day of their CI activation. The same stimuli were presented as in adults. The proportion of children where fNIRS was able to detect cortical responses was lower than in adults (100% in adults vs. 76–82% in children), but significant brain responses were found in all three groups of children. Moreover, similar hemodynamic responses were found in all the groups, adults and children, demonstrating that fNIRS is a reliable tool to measure activity in the auditory cortex in response to speech even in CI children, as early as the day of CI activation.

Coëz et al. (2022) aimed to better characterize the critical period before which CI must be implanted for optimal outcomes by looking at the activation of the auditory cortex in response to human voice and non-voice stimuli in young infants with and without hearing loss. Two groups of 12-month-old infants were tested: a group of deaf infants tested before CI surgery and a control group of normal-hearing infants. The voice condition consisted of speech stimuli like words, non-words, foreign languages, as well as non-speech stimuli like laughs, singing, or sighs. The non-voice condition contained natural sounds, animal sounds, environmental noises, and musical instruments. Participants’ brain activity was recorded using fNIRS targeting the bilateral temporal, frontal, and parietal cortices, where a human voice area had previously been identified in typically developing hearing 7-month-old infants (Grossmann et al., 2010). Coëz et al. (2022) found greater responses to the human voice stimuli than to non-voice sounds in deaf infants, but interestingly not in typically developing infants. Also, while typically hearing infants showed inverted hemodynamic responses, that is, a decrease in oxyhemoglobin and an increase in deoxyhemoglobin, deaf infants showed a canonical response to the human voice. Furthermore, deaf infants showed a significant correlation between the cortical responses to the voice condition and their residual hearing threshold at 250 Hz, which roughly corresponds to the fundamental frequency of the human voice. Taken together, these results suggest that by 12 months of age, typical infants show a rapid habituation, that is, an inverted response, to these frequently encountered stimuli, the processing of which are effortless and metabolically undemanding, thus showing no difference any more between conditions, whereas their deaf peers exhibit a strong response to the human voice, similarly to typically developing infants at an earlier age. Furthermore, these results, as those of Sevy et al. (2010), suggest that fNIRS is a reliable tool to measure the cortical responses of deaf infants and children to auditory stimuli. It is also interesting to note that Coëz et al. (2022) followed up the two groups at 24 and 36 months of age, that is, 12 and 24 months after implantation. They did not obtain usable data in sufficient numbers of implanted children, in part due to difficulties combining the fNIRS cap with the CI; thus, they describe the challenges of fNIRS capping in the presence of a bilateral or unilateral CI, and provide recommendations for efficient capping procedures on the basis of their experience.

In contrast, Wang et al. (2021) successfully conducted fNIRS measurements both before and after CI surgery. They tested 22 prelingually deaf infants and toddlers (aged 13–30 months), 1 week before surgery and again within a week after CI activation. Participants were exposed to four vocal emotional stimuli (pseudo-sentences with varying emotional contours: fear, anger, happiness, and neutral). The neural responses differed significantly between the preoperative and postoperative fNIRS tests, with the postoperative activation resembling more typical neural processing. Specifically, the pre-motor and the supplementary motor cortex in the left hemisphere shifted from preoperative activation to postoperative deactivation, while the middle temporal gyrus, and the superior temporal gyrus in the right hemisphere showed the opposite pattern, from preoperative deactivation to postoperative activation. These patterns were consistent across the four emotional conditions, suggesting that even after surgery, children did not effectively discriminate between them. Only the right supramarginal gyrus showed a stronger response to fear compared to anger. In contrast, normal-hearing newborns could recognize a broader range of emotional stimuli, demonstrating more advanced processing than children tested at CI activation. These findings suggest that CI triggers a rapid change in the processing of vocal emotions, even if such processing does not yet resemble the one observed in normal-hearing newborns.

In a study investigating auditory processing after CI, Y. Chen et al. (2022) presented natural utterances with strong (SP) or weak (WP) prosodic features to 5–10-year-old children with prelingual deafness corrected with unilateral CI (n = 21) and to 5–7-year-old normal hearing (NH) controls (n = 25). WP sentences were regularly pronounced sentences, while SP sentences had rhyming words and a strong rhythm. SP sentences elicited broad, right-lateralized cortical responses compared to WP stimuli in NH children. The CI group showed a significantly weaker activation in response to both conditions, with less activation to SP than to WP, suggesting an altered/immature sensitivity to strong prosodic features of speech. Importantly, CI children had at least 12 months of CI experience, but the age of implantation (AoI) ranged between 1.5 to 9.7 years, reflecting variability in the duration of auditory deprivation. The authors, however, did not enter this variable into the analysis, so its significance cannot be assessed.

Another group found somewhat different results. Z. Chen et al. (2024) used both classification and individualized regression models to predict behavioral improvement after CI from fNIRS data. Neural data were collected from 47 children (35.47 ± 17.24 months) within the first months after CI activation and then at 12.11 ± 9.62 months after CI activation. The auditory paradigm included speech, noise, music, and speech in noise stimuli. Three questionnaires were administered to participants’ caregivers to measure participants’ auditory and speech perception abilities. Through machine learning, fNIRS data collected shortly after CI activation accurately predicted later behavioral improvements, with the highest accuracy yielded by hemoglobin changes in response to more than two sound conditions. Interestingly, audiological and demographic factors (e.g., AoI, side of implantation, residual hearing in CI side and non-CI side, gender, duration of CI experiences, duration of hearing aid experiences) did not provide reliable improvement in classification accuracy.

In general, the studies that presented children with different voice conditions (and non-voice ones, as in Coëz et al. (2022) and Z. Chen et al. (2024)) found immature auditory processing both before (Coëz et al., 2022) and after (Y. Chen et al., 2022; Wang et al., 2021) cochlear implantation: DHH children’s responses were typical, but delayed, similar to the responses of *younger* normal hearing children. We note, however, that in the reported studies, the samples were highly variable in chronological age and age of implantation. For instance, in Coëz et al. (2022), subjects were implanted at 12 months, while those in Y. Chen et al. (2022), Z. Chen et al. (2024), and Wang et al. (2021) at an age varying between 1–9 years. This difference affects the duration of auditory deprivation, which can lead to cross-modal cortical changes, whereby sensory brain regions deprived of input may be recruited to perform non-auditory functions (Bavelier & Neville, 2002). We now turn to addressing this issue in developmental populations.

### 2.2 Neural reorganization as a result of auditory deprivation and the subsequent restoration of hearing

How auditory deprivation shapes the brain has been a central question in the last decades. Results with adults are conflicting. It is unclear whether neural reorganization occurs after acquired hearing loss and whether plastic changes in the brain are advantageous or detrimental to language processing after the restoration of hearing with CI or other devices (Anderson, Wiggins, et al., 2017). Those who support the hypothesis of adaptive neuroplasticity claim that this reorganization is beneficial, because it enhances speech understanding after cochlear implantation, possibly by keeping the brain areas active despite deprivation (Anderson et al., 2019; Fullerton et al., 2023). Those who support the hypothesis that neuroplastic changes are maladaptive argue that these changes interfere with speech processing after CI as they channel neural resources away from auditory processing (Doucet et al., 2006; Sandmann et al., 2012).

Since neuroplasticity is at its height early in life, investigating these plastic changes in children may provide relevant insight into the debate. We, thus, discuss fNIRS studies investigating neural reorganization across sensory modalities in pediatric populations with congenital or early-onset hearing loss in response to audiovisual or visual-only stimuli.

Mushtaq et al. (2020) examined the influence of cross-modal plasticity on speech comprehension in a sample of children with CI, with good perceptual skills, and in normal hearing controls (NH) of the same age (6–12 years). Through an event-related paradigm, they exposed children to sentences presented in four experimental conditions: visual speech for lip-reading, normal auditory speech (modulated and intelligible), signal-correlated noise (SCN, modulated but not intelligible), and speech-shaped noise (SSSN, unmodulated and not intelligible). The hemodynamic responses to auditory stimuli did not differ between the two groups, nor in the other contrasts of interest (speech vs. SCN and SCN vs. SSSN), indicating a considerable overlap in functional brain patterns to auditory stimuli, even if CI users presented a greater number of activated channels. Conversely, the two groups significantly differed in the visual condition in which CI children demonstrated greater activation in the superior temporal cortex in both cerebral hemispheres. Differently from the maladaptive plasticity effects found in adults, in this group of CI children the perceptual abilities were generally good, suggesting either that cross-modal plasticity is adaptive for the successful development of auditory speech perception following cochlear implantation, or at least that it leaves auditory processing unaltered. Similar results were found later by X.-Q. Zhou et al. (2023), who presented 6–12-year-old CI users and controls with spoken sentences in quiet, in noise, and only visually (for lip-reading). Hemodynamic responses in the superior temporal gyrus were on average greater in the CI children compared to controls. Nonetheless, the two groups did not significantly differ in the two auditory conditions, while they did differ significantly in the visual condition. Besides, better speech comprehension was associated with stronger activation in response to visual speech in this ROI in the CI children, suggesting that greater recruitment of auditory brain regions for visual speech processing would facilitate the restoration of hearing after implantation. Lastly, Alemi et al. (2023) investigated the audiovisual integration of CI children using both EEG and fNIRS. To do so, they tested seventy-five children aged 7 to 18 years, separated in two groups: a group of 50 children with CI and a control group of 25 children with normal hearing. The group of CI children was also divided into two subgroups: a group of 26 children with age-appropriate language skills and a group of 24 children with language delays. The stimuli consisted of two syllables, /ba/ and /ga/, which differ in their place of production. When presented visually, the syllable /ba/, which contains a bilabial consonant, has a completely clear visual cue, while /ga/ comprises a back consonant with no visual cue to its place of articulation. The stimuli were presented in three conditions: audio-only, visual-only, and audio-visual. EEG and fNIRS were recorded sequentially with two different paradigms corresponding to the time constraints of each technique (fast with a high number of trials for EEG, slow, but with fewer trials for fNIRS). In the EEG paradigm, stimuli were repeated 30 times for each condition, while for fNIRS, the syllables were presented in a block design. The three groups did not differ significantly. In particular, the fNIRS data revealed that the visual (V) and audiovisual (AV) conditions triggered a significant activation of the visual cortex while the auditory (A) condition led to a significant deactivation of it. The auditory regions (Wernicke’s area and inferior parietal lobules, IPL) were activated by the auditory condition, deactivated by the visual condition, and did not show any significant change during the audio-visual presentation. Interestingly, the activation of the Wernicke’s area and the IPLs was inversely correlated to the activation of the auditory cortex. The EEG results were congruent with the fNIRS ones, showing a strong activation in the occipital area in response to the V and AV conditions but not to the A condition. The authors suggest that the deactivation of the visual cortex during non-visual tasks may be an index of healthy cognitive processing. Once again, the results are in line with the adaptive hypothesis.

The same research group tested the same participants in other tasks presenting visual-only stimuli (Alemi et al., 2024; Deroche et al., 2024). Deroche et al. (2024) aimed to investigate how the brains of 7–18-year-old children, who were implanted early, responded to low-level visual cues. They presented CI children and age-matched controls with a circular checkerboard rotating while recording their brain activity both with high-density EEG and whole-head fNIRS in two separate sessions. In the EEG task, CI children with language delays showed weaker visual cortex responses compared to CI children with age-appropriate language skills and normal hearing controls. The response during the visual task was synchronized with a modest activity over auditory association areas in controls and CI children with age-appropriate language skills. By contrast, this synchronized activity was progressively lost with later age of implantation, which was characteristic of the CI group with language delays. Furthermore, the hemodynamic responses in the occipital ROI did not differ between the groups, while the ones in the temporal ROI did. Controls consistently deactivated the superior temporal gyrus and middle temporal gyrus, whereas CI children did not present this inverted response. The authors interpreted these results as a failure to inhibit the auditory association areas in the CI children. The difference in activation between the CI and the control groups was due to the inverted response in the latter group. The authors interpreted the deactivation of the temporal areas as a sign of disengagement of the auditory areas during visual tasks in the control children, which they consider adaptive. CI children’s failure to show a similar disengagement may, thus, be regarded as maladaptive. Thus, the authors interpret both the EEG and fNIRS findings as supportive of the maladaptive hypothesis, since auditory deprivation did not result in a more efficient visual system, especially for CI users with delayed language. It must be noted that the stimuli (visual speech vs. low-level visual processing) are different, and the age range is wider in this study than in most others discussed above (Mushtaq et al., 2020; X.-Q. Zhou et al., 2023).

The same participants were also tested in another task reported in Alemi et al. (2024). They investigated motor processing in the CI children, in order to better understand if deafness impacts the auditory-motor and/or visual-motor networks, which are often recruited in daily life. The participants had to perform a simple and repetitive motor task, they had to press a joystick button each time the word “squeeze” was displayed on the screen, while their brain activity was recorded in four regions of interest: the motor cortex, the somatosensory cortex, the visual cortex, and the superior temporal gyrus. fNIRS was able to reveal a significant activation of the motor cortex during the task in all children. No significant activation was found in the somatosensory cortex nor in the superior temporal gyrus. A significant effect of group was found in the visual cortex, explained by a larger deactivation in CI children with good language skills compared to CI children with language delays. The authors suggested that this deactivation of irrelevant brain regions during a task in the group with good language skills may be related to a resource allocation strategy process providing more cognitive resources to the relevant brain regions. Discussing their findings, the authors pointed to limitations in the use of fNIRS. One-third of the participants showed very little hemodynamic activity, suggesting that fNIRS may not always be able to track cortical activity, due, for instance, to hair or skin-related factors. Moreover, the authors questioned the fact that fNIRS is able to measure certain cortical regions, like the primary auditory cortex, which is situated deep in the sylvian fissure, possibly beyond the penetration depth of fNIRS.

In summary, hemodynamic responses have been observed in the temporal areas during visual tasks in children and adolescents with cochlear implants (Mushtaq et al., 2020; X.-Q. Zhou et al., 2023). Their interpretation remains unsettled, as they can be seen as adaptive, supporting plastic changes, or maladaptive since typically hearing children often present a deactivation in this situation. It is also noteworthy that in these studies, the deaf groups showed similar hemodynamic responses to auditory stimuli as controls (Alemi et al., 2023; Mushtaq et al., 2020; X.-Q. Zhou et al., 2023). This seems to partially contradict the results found in auditory-only studies, which have shown more immature patterns in response to several types of auditory stimuli. However, except for Y. Chen et al. (2022), the previously described auditory-only studies tested younger children (below 5 years of age), while the participants in the auditory-visual and visual studies were older (all above 6 years and up to 18).

### 2.3 Neural network connectivity in DHH and CI children

Some fNIRS studies have investigated how deafness and CI change the development of neural networks and connectivity in children with bilateral (G. Liu et al., 2023; Tan et al., 2024; Wu et al., 2024, 2025) and unilateral hearing loss (Calmels et al., 2022; Y. Liu et al., 2023), as well as CI (Wang et al., 2022).

G. Liu et al. (2023) investigated functional connectivity during resting state in 3–9-month-old infants with sensorineural hearing loss and age-matched controls. They found no significant differences between the two groups in the small-world organization of their neural networks, suggesting that the capability of local specialization and global integration within brain networks has not yet been disrupted in young infants with congenital hearing loss. However, while normal hearing infants showed increased asymmetry between the left and right hemispheric networks’ efficiency with age, the HL group did not, independently of the degree of the hearing loss, suggesting that even mild hearing loss could disrupt the development of cerebral asymmetry.

Similar findings were observed in Tan et al. (2024), who compared 4-month-old infants with sensorineural hearing loss to both NH newborns and NH 4-month-old infants. Functional connectivity patterns differed across the three groups, with the older NH group showing the most enhanced functional organization and integration. Conversely, the HL group seemed to exhibit neural dysfunction and different developmental trajectories, likely due to hearing loss.

In studies by Wu et al. (2024, 2025), older CI children’s brain activity was recorded during resting state and passive listening to speech and noise. The data were collected first shortly after CI activation, then up to 3 years after activation. Wu et al. (2025) showed that CI children exhibited stronger intra- than interhemispheric connectivity, along with greater immaturity in temporo-parietal connections than temporo-frontal ones. The left temporo-fronto-parietal network developed faster than its right counterpart. The authors also found a function-specific differentiation of cortical networks with early hearing experience, since the language network and the resting-state networks had different developmental trajectories. Importantly, developmental changes for both rest and speech predicted later auditory performance.

These predictive findings were further validated in Wu et al. (2024), who used a support vector machine (SVM) classifier to predict post-implantation improvement from cortical responses at CI activation. They found an initial advantage of speech over noise processing during the first 2 months of hearing experience, though this advantage diminished in about 1 year.

Wang et al. (2022) focused on side of implantation by investigating children who received a unilateral CI. Children were tested at least twice within the first 6 months after surgery and their brain activity was recorded both during rest and while listening (speech, music and noise). The hemodynamic responses to speech showed no influence of implantation side, nor developmental changes. By contrast, children with left CI demonstrated better discrimination of nonspeech sounds (music vs. noise), and their responses in the right anterior temporal lobe were correlated with the age of implantation, providing a possible neural mechanism for the often-observed advantage of early implantation.

Lastly, Y. Liu et al. (2023) investigated resting-state activity in 3–10-month-old infants with unilateral hearing loss (UHL) and controls. UHL refers to hearing loss in one of the two ears, when the other ear has normal hearing. When UHL is profound, it is often referred to as single-sided deafness (SSD). Clinical practice is highly variable regarding the treatment of UHL and SSD, and as a result the usefulness of CI in SSD patients is not fully understood. Although SSD children do not experience auditory deprivation to the same extent as those with bilateral hearing loss, SSD still affects auditory development and its neural correlates (Gordon et al., 2013; Kral et al., 2013). Y. Liu et al. (2023) found no differences in functional connectivity between the two groups. However, within the UHL group, infants with SSD actually showed increased functional connectivity, especially in the right hemisphere, irrespective of the side of hearing loss. Similarly, Calmels et al. (2022) proposed a research protocol which aims at investigating the functional reorganization of auditory asymmetry in SSD children. The authors suggest using fNIRS to measure the activation of the auditory cortex in response to different types of sounds (environmental sounds, voiced speech and non-speech sounds, and white noise) in non-implanted children with SSD and assess whether these responses predict auditory and language skills.

In summary, these studies showed how early the effects of hearing loss, including compensatory reorganization, begin to emerge in cortical networks (G. Liu et al., 2023; Y. Liu et al., 2023; Tan et al., 2024) and how the brain reorganizes after cochlear implantation (Wu et al., 2024, 2025), highlighting that cortical responses at CI activation may be predictive of later outcomes (Wu et al., 2024, 2025), and underlining the advantage of early implantation (Wang et al., 2022).

## 3 Methodological Challenges

The above discussed studies firmly establish that fNIRS is a useful and promising new tool for investigating how deafness, language deprivation, and the restoration of hearing after cochlear implantation shape the developing brain. However, methodological challenges remain.

Researchers face a number of challenges when using fNIRS with cochlear implanted children above and beyond those present for developmental neuroimaging. First, the fNIRS headgear needs to accommodate the implant (Sevy et al., 2010). As most headgears take the form of a closed, stretchy cap or headband wrapped around the head, positioning them on top of the implant can cause the optodes surrounding the implant site to lift up and lose contact with the skull, compromising signal quality. Additionally, the placement of the headgear may cause the processor to detach from the receiver, impairing the proper functioning of the implant. Moreover, the cap/headband may cover the microphone and compromise the quality of the perceived speech signal. Since the implant is located close to the temporal and parietal cortices, both of which are of particular relevance for speech and language processing, this problem needs to be solved for successful fNIRS recording. Headgears, thus, need to be designed or custom-made with the implant in mind, for example, by cutting a small aperture in the cap/band for it. This is a methodological challenge that researchers have direct control over, having the possibility to choose headgear designs that allowed them to improve and maximize data quality.

Finding the appropriate stimuli and paradigm, especially in the pre-implantation and the early post-implantation phases, when children do not yet hear sufficiently well, is not easy, as the auditory stimuli may not capture participants’ attention well enough to trigger a response and to keep them still and focused on the task. This results in poor data quality, movement artifacts, and a low signal-to-noise ratio. It is, therefore, necessary to adjust the volume of the stimuli appropriately and to use visual attention getters, for example, a silent movie or cartoon, silent toys, puppets, and so on to keep infants and young children on the task, even if they cannot hear the auditory stimuli well or perceive them as uninterpretable and thus uninteresting.

Another methodological challenge derives from the fact that fNIRS can only probe the (surface of the) cortex. With a distance of 3–4 cm between light sources and detectors, typically used in most commercial systems, the penetration depth is around 3 cm from the scalp into the head, corresponding to about 1–1.5 cm into the cortex in infants, and about 0.5 cm in adults whose skin, skull, and other tissues are thicker (Delpy & Cope, 1997; Hiraoka et al., 1993). This penetration depth is sufficient to reach a large number of the speech- and language-related areas in the temporal, parietal, and frontal cortices at least in young infants, but many other cortical areas and most subcortical structures remain invisible, especially in older children, as discussed in Alemi et al. (2023). This limits the uses of fNIRS to perceptual and cognitive functions whose neural bases are located sufficiently close to the surface of the cortex.

Using fNIRS with developmental populations is also challenging because the hemodynamic response changes as a function of development. When fNIRS is used with DHH or CI developmental populations, changes seen in the HRF over time confound physiological changes due to biological maturation and changes related to experience (e.g., duration of deprivation, activation of CI, or duration of CI use). Experimental designs should, thus, be careful to unconfound these factors, for example, by using controls for biological maturation as well as controls for auditory or language experience.

Possibly the greatest challenge when using fNIRS with cochlear implantees or other clinical populations is the question of whether fNIRS measures are reliable at the individual level. While even just a decade ago, fNIRS was mainly only used to draw conclusions at the group level, for example, to establish a difference in brain activation between a pathological group and healthy controls, recent advances are increasingly making fNIRS a suitable tool for individual-level assessment of neural activation, sufficiently reliable for personalized clinical applications such as Brain-Computer Interfaces in vegetative state patients (Abdalmalak, 2020; Kazazian et al., 2024) or classifying stress-tolerance or expertise in surgeons performing surgery (Leff et al., 2008; Modi et al., 2017). NIRS is usually not yet used alone as the sole diagnostic tool in clinical practice, but in combination with other measures, it is increasingly relied upon to establish a diagnosis, or to monitor the efficiency of an intervention. Due to its ease of use, non-invasiveness, and low operation costs, fNIRS is particularly well-suited for repeated assessments or long-term monitoring, which, for instance, MRI does not easily allow. As suggested by work on adults (Olds et al., 2016), fNIRS can be a powerful tool for personalized, individual-level assessment of cochlear implanted children’s language development, especially in combination with other measures such as audiometry, language, and vocabulary development questionnaires etc. Long-term fNIRS monitoring of cochlear implant success will be particularly easy when wearable fNIRS devices, the current trend in fNIRS hardware development (Ayaz et al., 2022; Pinti et al., 2018; Vidal-Rosas et al., 2023), become routinely used. With wearable devices, it will become possible to test implantees’ brain responses in ecologically valid, real-life situations that are especially difficult for them, for example, perceiving speech in noise, using a foreign language, or playing music. An implanted child may thus be wearing an fNIRS device while in daycare or at school, or when taking a piano lesson.

## 4 Open Questions and Future Prospects

With these methodological issues solved, fNIRS has the potential to help answer the critical open questions about the impact of deafness and cochlear implantation on the brain. Most importantly, fNIRS could provide useful insight about individual differences in brain plasticity and the extent to which these contribute to inter-individual variation in CI outcomes. Is it the case that individuals with greater brain plasticity learn to use their CIs with greater ease and efficiency? Relatedly, do we see critical period effects in CI users? It is particularly relevant to investigate the youngest infants, as recent work on critical periods in typically developing children suggests that language experience received as early as 3–5 months of age may have a lasting impact on the brain (Choi, Broersma, et al., 2017; Choi, Cutler, et al., 2017). Indeed, international adoptees showed perceptual saving from the birth language even when they were adopted at age 3–5 months, that is, before perceptual narrowing to the phonology of the native language takes place. In the light of these and other results on critical periods (Reh et al., 2020; Werker & Hensch, 2015), it is crucial to understand how deafness and language deprivation impact brain development. On the basis of other critical period phenomena, at least two scenarios are possible. Language deprivation, especially extended periods of it, may be detrimental to later language outcomes, as the critical period for language acquisition may already be closed, as data from typically developing infants and international adoptees may suggest (Choi, Broersma, et al., 2017; Kuhl et al., 1992; Werker & Hensch, 2015; Werker & Tees, 1984). Alternatively, auditory and language deprivation may delay the onset of critical periods, as is the case, for instance, in the visual development of dark-reared animals (Hensch, 2005) and to some extent in children with binocular cataracts (Maurer et al., 1989). In this case, the learning opportunity may not be lost, and the critical period may open when language experience begins. Investigating implanted children’s brain plasticity may help us adjudicate between these alternatives. Previous results have clearly established that earlier implantation leads to better outcomes (Kral & Sharma, 2012; Sharma et al., 2009, 2013). Indeed, children implanted before 3–5 years show normal or close to normal electrophysiological responses to speech sounds, while those implanted later often do not catch up completely with their hearing peers. But even among children implanted early, there are differences in outcome, and fNIRS can shed light on whether differences in brain plasticity between CI users may be a factor behind this variability. NIRS is a useful tool to address these questions, testing deaf children’s brain not only after, but even before implantation.

As the flipside of the same coin, fNIRS can help us better understand whether and if yes, to what extent, deaf infants’ brains undergo reorganization due to deprivation. Are auditory areas recruited by visual or other sensory modalities in the absence of auditory input, as suggested by the maladaptive hypothesis proposed for adults (Anderson, Wiggins, et al., 2017)? Are there differences in this regard between deaf infants who receive language in the visual modality (e.g., sign language) and those who do not? Do either of these influence CI outcomes after implantation? By investigating deaf and cochlear implanted infants’ brain responses to auditory and visual stimuli, fNIRS can help us address these questions.

NIRS can also play an important role in mapping deaf and hard-of-hearing children’s residual auditory and speech perception abilities. Relying on their residual hearing, deaf infants may be sensitive to more linguistic contrasts than currently believed, for example, as suggested by their sensitivity to the voice/no-voice distinction (Coëz et al., 2022), but these abilities are usually not mapped. This deprives parents and clinicians of highly relevant information, as these abilities could be relied up for interventions, therapy and rehabilitation already before, as well as after implantation.

Moreover, fNIRS could be a frontier technique for the evaluation and treatment of children with unilateral hearing loss. Few studies exist on this population (Gordon et al., 2023). fNIRS research can help better understand the impact of an early intervention program UHL children’s early cortical development. fNIRS evaluation can be a useful tool to promote personalized auditory habilitation programs and to prevent long-term neurofunctional and clinical consequences considering that UHL seems to affect not only auditory functions but also speech and language development, as well as cognitive functions.

## 5 Conclusion

In summary, the existing literature on the neural mechanisms triggered by auditory and language deprivation in deafness and the neural changes induced by the restoration of hearing after CI is incomplete, with studies varying in methods, population characteristics, measures, and so on. Further studies are needed to systematically explore the relevant factors. fNIRS has proven to be a powerful tool to address these issues. The existing studies are beginning to reveal a complex interaction between different factors such as experience and brain plasticity. fNIRS also allows researchers and clinicians to map perceptual abilities before and after implantation in order to monitor progress, identify residual abilities that can be built upon during rehabilitation, and recognize early signs of difficulties much before delayed production abilities and poor cognitive or educational outcomes become apparent, sometimes years after implantation. fNIRS, thus, offers an early window of opportunity for successful intervention in CI children.

## Ethics

This paper does not report a study testing human participants.

## Data and Code Availability

No datasets or code are associated with this paper.

## Author Contributions

Conceptualization: A.R.F., J.G., and P.T. Writing—original draft preparation: J.G., G.L., and C.N. Writing—review and editing: D.B., V.D.V, A.R.F., J.G., G.L., A.M., C.N., and P.T. Supervision: J.G. Funding acquisition: A. R.F., J.G., and P.T. All authors have read and agreed to the published version of the manuscript.

## Funding

This work was supported by the “SYNPHONIA” (nr. CLP: PNRR-MAD 2022-12376739; CUP: C63C22001320007) project funded by the European Union Next Generation EU (NRRP M6C2, Investment 2.1 “Enhancement and strengthening of biomedical research in the NHS” (A.R.F., P.T.), the ERC Consolidator Grant “BabyRhythm” no. 773202 (J.G.), a FARE grant nr. R204MPRHKE from the Italian Ministry for Universities and Research, the PRIN grant nr. 2022WX3FM5 from the Italian Ministry for Universities and Research (J.G.), and BRIC INAIL 2022 ID 08-2022 nr. 470 (A.R.F.).

## Declaration of Competing Interest

The authors declare no conflict of interest.
